# Facteurs de risque de faible poids de naissance en milieu semi-rural de Kamina, République Démocratique du Congo

**DOI:** 10.11604/pamj.2014.17.220.2366

**Published:** 2014-03-20

**Authors:** Ignace Bwana Kangulu, Elie Kilolo Ngoy Umba, Michel Kabamba Nzaji, Prosper Kalenga Mwenze Kayamba

**Affiliations:** 1 Université de Kamina, Faculté de Médecine, Département de Gynécologie et Obstétrique, Kamina, République Démocratique du Congo; 2 Université de Kamina, Faculté de Médecine, Département de Santé Publique, Kamina, République Démocratique du Congo; 3 Université de Lubumbashi, Faculté de Médecine, Département de Gynécologie et Obstétrique, Lubumbashi, République Démocratique du Congo

**Keywords:** Facteurs de risque, faible poids de naissance, Kamina, risk factors, low birth weight, Kamina

## Abstract

**Introduction:**

Le faible poids de naissance constitue un problème majeur de santé publique, aussi bien dans les pays développés que dans les pays en développement, de par son ampleur et sa forte association avec la morbidité et la mortalité infantiles.

**Méthodes:**

Il s’agit d’une étude cas-témoins rétrospective menée sur les différents facteurs de risque de faible poids de naissance(FPN) en milieu semi-rural de Kamina chez les accouchées et leurs nouveau-nés respectifs enregistrés de la période allant de janvier 2009 à décembre 2010.

**Résultats:**

Cette étude a répertorié 69 cas des nouveau-nés de FPN sur 483 accouchements enregistrés (14,3%). Les facteurs associés au FPN déterminés dans ce travail sont l’âge maternel inférieur à 18 ans (OR=7,62, IC=3.46-16.8) et supérieur à 35 ans (OR=2,04;IC=0.91-4.46), la primiparité(OR=2,48;IC=1.18-5.21) et le non suivi des consultations prénatales (OR=5,50;IC=2.00-15.03), la prématurité avec grossesse âgée de moins de 37 semaines d’aménorrhée, la grossesse multiple (OR=30,94) et le sexe féminin du nouveau-né.

**Conclusion:**

Cette étude a démontré que l’âge maternel inférieur à 18 ans et supérieur à 35 ans, le non suivi des consultations prénatales, la primiparité et la prématurité, la grossesse multiple et le sexe féminin du nouveau-né sont les facteurs associés au faible poids de naissance.

## Introduction

Le faible poids de naissance (FPN) est défini par l’Organisation Mondiale de la Santé (OMS), comme un poids à la naissance strictement inférieur à 2500g, quel que soit le terme de la grossesse [1]. Il constitue un problème majeur de santé publique, aussi bien dans les pays développés que dans les pays en développement, de par son ampleur et sa forte association avec la morbidité et la mortalité infantiles. En 2004, d’après les estimations de l’UNICEF, plus de 20 millions d’enfants sont nés avec un FPN dans le monde entier, ce qui représente 15,5% de l’ensemble des naissances; la plupart de ces naissances de faible poids (96%) ont lieu dans les pays en développement. Dans ces pays, la proportion des FPN (16%) est le double de celle des pays développés [2].

Le poids de naissance est donc un important indicateur de l’état de santé et de la situation nutritionnelle de la mère avant et pendant la grossesse. C’est aussi un important prédicteur de la survie de l’enfant et de son développement ultérieur [3]. Il y a en effet une association étroite à court terme entre le niveau de FPN, la mortalité fœtale et néonatale et la morbidité infantile [4, 5]. Parmi les 11,6 millions de décès d’enfants de moins de 5 ans survenus en 1995 dans les pays en développement, 6,3 millions (soit 53%) étaient associés au faible poids de naissance [6]. A moyen terme, le FPN est associé à un déficit de développement cognitif et physique avec réduction des capacités intellectuelles de l’enfant [7].

Ces enfants sont en outre prédisposés aux pathologies chroniques et cardiovasculaires liées à l’alimentation à l’âge adulte [8]. Par ailleurs, la prise en charge par le système de santé des pays en développement des enfants nés avec un déficit de croissance est en général insuffisante ou inadéquate, en raison de son coût élevé. Il en découle alors des conséquences importantes pour les sociétés, en termes de pertes en capital humain et en productivité économique. Les causes et les conséquences du FPN sont complexes et jouent un rôle important dans le cycle de vie de l’individu; au centre de ce cycle, se trouve l’environnement nutritionnel intra-utérin, déterminant important de l’état de santé et de croissance ultérieur d’un individu.

Afin d’identi’er les facteurs de risque obstétricaux associés au petit poids de naissance en milieu rural sahélien, Patrick Kabore et al ont mené une étude transversale au Centre Nord du Burkina et ont répertorié 1013 nouveau-nés d’une grossesse unique à terme. Après ajustement pour les facteurs socio-économiques, la primiparité (OR=2,8), les vomissements gravidiques (OR=3,4), l’exécution de travaux champêtres (OR=3,3) et une charge de travail élevée en cours de grossesse (OR=1,6) ainsi que l’accouchement à domicile sans assistance (OR=2,1) étaient les facteurs significativement associés au petit poids de naissance même si le nombre de consultations prénatales ne confère aucun avantage préventif vis-à-vis de la survenue du déficit pondéral à la naissance. L’étude a montré la nécessité d’une redéfinition du contenu et des procédures du suivi de la grossesse incluant une prise en charge adéquate des vomissements gravidiques et de sensibilisation des populations sur l’allègement de la charge de travail des femmes enceintes [9].

Une autre étude cas témoin a été réalisée dans 8 maternités de Ouagadougou du 30 mars au 10 juin 2005, afin d’évaluer l’importance des facteurs d’origine alimentaire et nutritionnelle associés au retard de croissance intra-utérin au Burkina Faso. A l ‘analyse univariée, les facteurs de risque significativement associés au retard de croissance intra-utérin(RCIU) étaient l’âge maternel < 20ans, la taille maternelle, l’IMC(indice de masse corporelle) avant accouchement, le gain de poids hebdomadaire, l’IMC après accouchement, le périmètre brachial < 24cm, le faible score de diversité alimentaire, l’insuffisance alimentaire ressentie par les femmes, le nombre de consultations prénatales < 3, le faible statut de la femme dans la société et le faible niveau socio-économique du ménage. A l’analyse multi variée, la petite taille (OR=2,13 (1,01-4,48)), le faible périmètre brachial (OR=3,02 (1,78-5,12)) et la consommation d’alcool pendant la grossesse (OR=2,89 (1,06-7,91)) restaient significativement associés au RCIU [10].

Le faible poids de naissance est un problème de santé multifactoriel dont la prévention est possible par des interventions ciblées sur des facteurs modifiables ayant fait leurs preuves d’efficacité dans plusieurs pays du monde. Ainsi, le taux d’incidence des faibles poids de naissance est l’un des indicateurs de la santé périnatale recommandé par l’OMS [11].

Tout ce qui précède nous montre de manière claire l’importance de mener des études sur les enfants venant au monde avec un déficit pondéral non seulement sur leur prévalence mais aussi et surtout sur les facteurs de risque d’origines maternelle et fœtale. C’est dans ce contexte que s’inscrit cette étude portant sur les facteurs de risque de faible poids de naissance dans le milieu urbano-rural de Kamina. La contribution à l’amélioration de la santé du couple mères-enfants et à la prévention des naissances d’enfants avec poids déficitaire dans le milieu urbano-rural de Kamina constituent l’objet principal poursuivi tandis que l’objectif spécifique du travail est celui d’analyser les différents facteurs de risque qui sont associés au faible poids de naissance.

## Méthodes

**Cadre d’étude**: La présente étude a été menée en République Démocratique du Congo dans la province du Katanga au centre de santé de référence BUMI, situé dans l’un des quartiers les plus peuplés de la cité de Kamina, le quartier Katuba I.

**Type, période et population d’étude**: Il s’agit d’une étude cas-témoin, portant sur les mères et les enfants nés durant la période allant du 1er Janvier 2009 au 31 Décembre 2010. Un cas a été défini comme tout nouveau-né dont le poids de naissance est inférieur à 2500g, mesuré à 10 grammes près sur un pèse-bébé mécanique. Un témoin était défini comme tout nouveau-né dont le poids de naissance était supérieur à 2500 g. Chaque cas a été apparié sur le même lieu d’enquête à six nouveau-nés de même sexe et de même âge (à 24h prés) nés à terme et pesant 2500 g ou plus.

**Critères d’inclusion et d’exclusion**: Sont inclus de cette étude tous les enfants nés vivants et leurs mères. Les variables étudiées ont été extraites des fiches de surveillance présentes à la maternité. Sont exclus de l’étude, les mort-nés et les couples mères-enfants ayant des dossiers ne contenant pas des renseignements sur les variables étudiées.

**Mode d’échantillonnage**: Nous avons examiné tous les dossiers des couples mères-enfants admis pour accouchement au centre de santé Bumi. C’est donc une étude exhaustive.

**Collecte et Analyse des données**: Les données ont été recueillies à l’aide d’une grille à partir des registres de la maternité du centre de santé de référence Bumi. Puis elles ont été encodées, saisies, traitées et analysées à l’aide du logiciel Epi-info.3.3.2 (version 2005) au seuil de 5%.

## Résultats

Les différents facteurs de risque de faible poids de naissance considérés dans la présente étude sont résumés dans Tableau 1. Il s’agit de l’âge de la mère, l’âge gestationnel, du suivi des consultations prénatales(CPN), de la parité et du type de grossesse. Dans ce tableau, deux types de nouveau-nés sont dégagés:ceux avec faible poids de naissance(FPN) et ceux avec un poids normal de naissance(PN).

**Tableau 1:** Répartition de poids de naissance selon les caractéristiques de la mère et de la grossesse

	Nouveau-né FPN	Nouveau-né PN	P	Odds ratio(OR)
Facteurs de risque	n(%)	n(%)		
**Age de la mère**				
< 18 ans	17(47,2%)	19(52,8%)	-	7,62 [3,46-16,8]
18-35 ans	41(10,5%)	349(89,5%)	-	1
>35 ans	11(19,3%)	46(80,7%)	0,000	2,04 [0,91-4,46]
**Age gestationnel**			**-**	**-**
< 37 SA	21(100,0%)	0(0,0%)		
≥37 SA	48(10,4%)	414(89,6%)	0,000	-
**Parité**			**-**	
Primipare	22(24,4%)	68(75,6%)		2,48 [1,18-5,21]
Paucipare	18(11,5%)	138(88,5%)	-	1
Multipare	29(12,2%)	208(87,8%)	0,0093	1,07 [0,55-2,09]
**Suivie de la CPN**			**_**¯	
Non	9(45,0%)	11(55,0%)		5,50 [2,00-15,03]
Oui	60(13,0%)	403(87,0%)	-	-
**Type de grossesse**				
Multiple	16(80,0%)	4(20,0%)	-	30,94
Monofoetale	53(11,4%)	410(88,6%)	0,00000	
**Total**	**69(14,3%)**	**414(85,7%)**	**-**	**-**

L’examen vertical du Tableau 1 permet de constater que sur total de 483 naissances enregistrées du 1er Janvier 2009 au 31 Décembre 2010 dans le Centre de Santé de Référence Bumi, 69(14,3%) concernent des nouveau-nés avec FPN et 414(85,7%) ceux nés avec un poids normal. En ce qui concerne les différents facteurs de risque soumis à nos investigations, il apparait que pour les âges maternel et gestationnel, les proportions FPN sont plus grandes chez les mères âgées de moins de 18 ans (OR=7,62) et lorsque la grossesse a eu moins de 37 semaines d’aménorrhée(SA). Dans le premier cas, 47,2% des cas ont été enregistrés et dans le second, 100% des cas. En outre, le Tableau 1 révèle qu’en matière des consultations prénatales(CPN) et de type de grossesse, les pourcentages de FPN se rapportent respectivement aux mères primipares (24,4%), aux gestantes qui n’ont pas suivi les consultations prénatales (45,0%) et aux grossesses multiples (80,0%). Signalons aussi que l’analyse des différents résultats permet de conclure statistiquement que ces différences sont significatives car elles sont toutes inférieures à la probabilité de 0,05(p inférieur à 0,05).

L’analyse du Tableau 2 montre que les nouveau-nés de sexe féminin sont plus prédisposés au faible poids de naissance avec une proportion de 19,1% que ceux de sexe masculin avec 9,9%(p=0,002).

**Tableau 2:** Répartition de poids de naissance des nouveau-nés selon leur genre

	Nouveau-né FPN	Nouveau-né PN	P
Genre du nouveau-né	n(%)	n(%)	
Féminin	44(19,1%)	186(80,9%)	0,002
Masculin	25(9,9%)	228(90,1%)	-
**Total**	**69(14,3%)**	**414(85,7%)**	**-**

## Discussion

Les proportions des nouveau-nés avec déficit pondéral sont de 42,2% chez les mères de moins de 18 ans, 10,5% chez celles de 18 à 35 ans et 19,3% après 35 ans. Cependant, comparativement aux gestantes âgées de 18 à 35 ans (moins exposées), celles de moins de 18 ans courent 7,62 fois plus le risque d’accoucher un nouveau-né avec faible poids de naissance. Ce risque devient de 2,04 chez les mères de plus de 35 ans. Il existe donc une association statistiquement significative (p=0,000) entre l’âge maternel (moins de 18 ans et plus de 35 ans) et l’accouchement d’un nouveau-né de petit poids de naissance. Ces résultats cheminent ensemble avec ceux de plusieurs autres auteurs [12–14].

En effet les adolescentes qui n’ont pas encore terminé leur propre croissance, sont plus susceptibles d’accoucher des enfants de faible poids à la naissance comparativement aux mères plus âgées ayant le même statut nutritionnel [15]. Cela pourrait s’expliquer par la compétition pour les nutriments entre l’adolescente en pleine croissance et le fœtus qui se développe ainsi que par la faible efficacité des fonctions placentaires à cet âge [16]. De plus la concurrence entre la grossesse et la croissance a un effet particulièrement défavorable sur le statut en micronutriment des adolescentes [17]. Ces mères adolescentes présentent souvent d’autres facteurs qui augmentent le risque d’accouchement d’enfants de faible poids à la naissance:race noire, niveau socio-économique faible, petite taille, faible niveau d’éducation, absence ou insuffisance de soins de santé prénatale [18]. Il semble de plus en plus évident que l’âge serait un facteur social de risque et non un facteur biologique, sauf chez les très jeunes adolescentes [19].

Concernant l’âge de la grossesse, nous avons noté que tous les nouveau-nés issus des grossesses de moins de 37 semaines d’aménorrhées ont donné naissance à des nouveau-nés avec faible poids (p=0,000). Après 37 semaines, seuls 10,4% des nouveau-nés sont concernés. Nos résultats sont comparables à ceux de la littérature [12, 20]. A ce propos, notons que le faible poids à la naissance résulte soit d’une naissance prématurée (accouchement avant la 37ème semaine d’aménorrhée), soit d’un retard de croissance fœtale des enfants nés à terme [21, 22]. Toutefois dans les pays où l’incidence du faible poids à la naissance est élevée (cas des pays en développement), ce dernier est principalement dû à un retard de croissance fœtale. Dans les régions où l’incidence est faible (cas des pays développés), la première cause se révèle être les naissances prématurées [3]. L’exposition des prématurés au déficit pondéral est expliqué par le fait que la naissance est intervenue à la période où le fœtus est encore en pleine croissance pendant la vie intra-utérine. Il est évident que la croissance fœtale dépend de la durée de la gestation. Une durée de gestation insuffisante ne permet pas au fœtus une croissance normale.

En matière des consultations prénatales, il a été constaté que les gestantes n’ayant pas suivi les CPN sont prédisposées à accoucher des nouveau-nés de FPN. Le risque chez ces femmes est évalué à 5,50. Cette constatation rejoint celle rapportée par d’autres chercheurs [12, 20]. Elle pourrait s’expliquer par le fait que le manque de suivi de la grossesse ne permet pas d’agir sur les causes médicales curables de faible poids de naissance ou de surveiller les résultats des mesures préventives systématiques contre le paludisme, l’anémie ou les carences nutritionnelles. De tels problèmes de santé publique sont courants dans le milieu de notre étude et se répercutent négativement sur la croissance pondérale du f’tus. Les soins prénatals et post-natals sont importants pour les femmes en âge de procréer, la surveillance prénatale permet de prévenir plusieurs maladies responsables de déficit pondéral à la naissance (paludisme, helminthiases intestinales, anémie.), la supplémentation en micronutriments (Fer, acide folique, vitamine A) et l’éducation de la femme enceinte sur l’hygiène de la grossesse. Le non suivi des CPN conduit facilement au FPN.

Considérant la parité, nous avons noté que les primipares courent 2,48 fois plus de risque de faible poids de naissance que les paucipares. Ce risque devient de 1,07 chez les multipares (à partir du 4è accouchement). La primiparité et la multiparité sont donc les facteurs de prédisposition au FPN comme l’ont constaté aussi plusieurs auteurs [12, 20]. Parlant de la primiparité, signalons que dans notre milieu d’étude, les primipares sont le plus souvent adolescentes en croissance. Le risque chez les multipares se justifie par le fait que les femmes en Afrique sub-saharienne passent la majorité de leur durée de vie, soit 35 à 50% des années de procréation, à répondre aux obligations de la grossesse, de l’accouchement et de l’allaitement [23]. Le syndrome d’épuisement maternel décrit ce qui se passe lorsque le corps de la femme est utilisé sans repos et sans temps pour récupérer [24]. On a observé que les coûts énergétiques de la grossesse et encore plus de la lactation, notamment dans le cadre de cycles de reproduction rapprochés (c’est-à-dire avec peu ou pas d’intervalle de récupération où la femme n’est ni enceinte ni allaitante), entraînent par un effet de cumulation, une dégradation de l’état nutritionnel maternel [25], pouvant ainsi conduire au FPN.

Nous avons aussi noté que 80% d’accouchements gémellaires ont donné des nouveau-nés avec petit poids de naissance et 11,4% seulement en cas de grossesse mono fœtale (OR=30,94). La différence statistique observée entre grossesse gémellaire et mono fœtale est très significative (p=0,00000). La littérature nous montre que les grossesses aboutissant à la naissance de jumeaux, triplés, ou plus sont à risque de faible poids à la naissance des nouveau-nés [26]. En effet, les femmes qui ont une grossesse multiple sont plus sujettes à l’hypertension et à l’anémie [27]. La gémellité constitue un facteur mécanique pouvant par ailleurs entrainer une insuffisance placentaire ou une transfusion foeto-fœtale [28]. En plus la sur distension utérine entrainée par la grossesse multiple cause le plus souvent un accouchement prématuré, donnant ainsi naissance à des enfants pesant moins de 2500g.

Enfin la répartition des nouveau-nés selon leur genre semble indiquer que ceux de sexe féminin sont plus atteints par le faible poids de naissance (19,1%° que ceux de sexe masculin (9,9%). La prédominance du sexe féminin chez les nouveau-nés de faible poids de naissance, concorde avec les données de la littérature [11]. Cependant, nous n’avons trouvé aucune raison expliquant cette prédominance féminine.

## Conclusion

Au terme de cette étude qui a permis d’établir les relations entre les facteurs maternels et fœtaux et le faible poids de naissance, Il a été démontré que l’âge maternel inférieur à 18 ans et supérieur à 35 ans, le non suivi des consultations prénatales, la primiparité et la prématurité, la grossesse multiple et le sexe féminin du nouveau-né sont les facteurs associés au faible poids de naissance. A l’exception de la primiparité, la grossesse multiple et le sexe féminin du nouveau-né, les autres facteurs sont modifiables et la mise en route d’un plan de réalisation des suggestions et des recommandations permettrait de les maitriser, de réduire les risques de faible poids de naissance et contribuer à la réduction du taux de mortalité infantile et à celle des complications lointaines dues à ce problème majeur de santé publique.

Cette étude a donc permis de montrer qu’outre les déterminants physiologiques non modifiables, certains déterminants importants restent accessibles. Des actions d’éducation et de sensibilisation bien ciblées et coordonnées sur la grossesse précoce, l’alimentation de la femme en âge de procréer de façon générale et de la femme enceinte en particulier pourraient avoir un impact positif sur l’amélioration du taux des naissances des enfants avec déficit pondéral. Toutefois, une étude complémentaire prospective dans la population générale serait nécessaire afin d’étudier d’autres facteurs et de mieux étudier les mécanismes par lesquels les différents facteurs s’enchaînent.
